# RFA Guardian: Comprehensive Simulation of Radiofrequency Ablation Treatment of Liver Tumors

**DOI:** 10.1038/s41598-017-18899-2

**Published:** 2018-01-15

**Authors:** Philip Voglreiter, Panchatcharam Mariappan, Mika Pollari, Ronan Flanagan, Roberto Blanco Sequeiros, Rupert Horst Portugaller, Jurgen Fütterer, Dieter Schmalstieg, Marina Kolesnik, Michael Moche

**Affiliations:** 1 Graz University of Technology, Faculty of Computer Science and Biomedical Engineering, Graz, 8010 Austria; 2 NUMA Engineering Services, Dundalk, 21222 Ireland; 3 Aalto University School of Science and Technology, Department of Computer Science, Espoo, 02150 Finland; 4 Turku University Hospital, Medical Imaging Centre of Southwest Finland, Turku, 20521 Finland; 5 Medical University of Graz, Division of Neuroradiology, Vascular and Interventional Radiology, Graz, 8010 Austria; 6 Radboud University Nijmegen, Radboud University Medical Centre, Nijmegen, 6525 Netherlands; 7 Fraunhofer Gesellschaft, Fraunhofer Institute for Applied Information Technology FIT, Sankt Augustin, 53754 Germany; 8 University Hospital Leipzig, Clinic for Diagnostic and Interventional Radiology, Leipzig, 04109 Germany

## Abstract

The RFA Guardian is a comprehensive application for high-performance patient-specific simulation of radiofrequency ablation of liver tumors. We address a wide range of usage scenarios. These include pre-interventional planning, sampling of the parameter space for uncertainty estimation, treatment evaluation and, in the worst case, failure analysis. The RFA Guardian is the first of its kind that exhibits sufficient performance for simulating treatment outcomes during the intervention. We achieve this by combining a large number of high-performance image processing, biomechanical simulation and visualization techniques into a generalized technical workflow. Further, we wrap the feature set into a single, integrated application, which exploits all available resources of standard consumer hardware, including massively parallel computing on graphics processing units. This allows us to predict or reproduce treatment outcomes on a single personal computer with high computational performance and high accuracy. The resulting low demand for infrastructure enables easy and cost-efficient integration into the clinical routine. We present a number of evaluation cases from the clinical practice where users performed the whole technical workflow from patient-specific modeling to final validation and highlight the opportunities arising from our fast, accurate prediction techniques.

## Introduction

Radiofrequency ablation (RFA) of liver malignancies has become an important alternative therapy for patients who disqualify for standard surgical treatment or are in an early tumor stage^1,2^. When surgical resection is not feasible, RFA is the preferred treatment option for small liver tumors^1,2^. Moreover, patient recovery after surgical resection takes longer and post-procedural quality of life is lower than after RFA^2^.

While many more options for local cancer treatment exist (*e.g*. Cryo Ablation^3^, Irreversible Electroporation^4^ or hyperthermia in conjunction with other treatment methods^5,6^), the clincial routine prefers RFA (or, occasionally, microwave ablation) treatment for smaller liver tumors. Although microwave ablation has become more prevalent in the past years, no statistically significant difference in survival rates compared to RFA of smaller lesions (diameter below 3.5 cm) in the liver could be found^7,8^.

In RFA, interventional radiologists (IR) destroy malignant cells using percutaneous probes that induce heating in a locally delimited region around a tumor. Successful treatment is defined as complete ablation of the tumor with a safety margin of destroyed healthy tissue in its immediate vicinity.

However, clinical experience with RFA indicates a significant mismatch between expected and observed lesion size, leading to reduced survival rates due to over-treatment with severe injuries (up to 9%) or under-treatment with tumor recurrence^9^ (up to 40%). Further, Hildebrand *et al*.^10^ have shown that the survival rates after 1 and 2 years significantly depend on the experience of the IR: Operating experience of 0–2 years resulted in 69%/46% (1/2 years) survival, while rates of 3–4 years experience corresponded to 92%/89%, respectively. Further, treatment of tumors larger than 3 cm is known to yield a higher local recurrence and a lower survival rate^11^ due to cumulation of unpredictabilities for larger lesions. This is strongly influenced by patient-specific factors, such as blood perfusion^12–14^, location of the tumor^15^, needle positioning^16–18^ and device-specific parameters, such as delivery of power or impedance^19,20^ and number of possible heating cycles^21^. In addition, continuous monitoring of the lesion growth during ablation is technically extremely challenging and therefore not clinical feasible to date.

Mispredicting the lesion size can lead to over- or under-treatment with specific risks to the patient. Simulating and visualizing the treatment outcome as observed one month post-ablation could help even experienced IRs to prospectively reinforce their decision making process. In general, IRs of any experience level can benefit from software-assisted planning and simulation of RFA in many scenarios. However, previous approaches for computational simulation of RFA^22,23^ are too inefficient and time-consuming for exploring the vast parameter space or only provide approximations^24^. Additionally, many parallel approaches require distributed computing to accelerate the simulation^25^, which can be difficult to integrate into clinical sites.

For intervention guidance, ultrasound (US) or computed tomography (CT) provide excellent visualization and control during placement of the RFA probe. However, these modalities do not offer *in situ* monitoring of the heat distribution. Therefore, treatment planning only involves manufacturer-specified heating protocols, which neglect patient-specific parameters. However, the heat sink effect of proximal vessels^26^ or the amount of porous tissue perfusion, affect the heat transfer and thereby shape and size of the lesion. Consequently, a simulation model adapted to the physiology can generate more accurate predictions. Therefore, it is desirable to use patient-specific data for treatment planning and modeling. Contrast-enhanced CT (ceCT) imaging allow determination of the vascular anatomy and quantification of the liver perfusion. Moreover, for accurate simulation of the thermally induced lesion around the RFA probe, its precise location *in situ* is essential. Breathing motion of the liver makes reproduction of pre-interventional planning almost impossible. This raises the need for co-registration of planned needle positions and images acquired during the intervention.

For the past two decades, researchers have utilized advanced technologies to visualize the internal anatomy of the body in 3D. Since the probe positioning plays a critical role in the RFA procedure^18,22,27–29^, a comprehensive visualization system containing both 2D and 3D views is important. Several RFA treatment planning and simulation environments have already been developed, for instance, a simulator and planner software solution for cryotherapy and its extension to RFA^30^. Further applications for RFA treatment planning exist utilizing different parameters spaces exist; e.g. RF-Sim tool^22^, MAXIO^31^, Robio^17^ and SAFIR^27,32^. GoSmart^33^ also features RFA simulations, but focuses more on providing a testbed for developers of hardware and software components and offering a communication platform between clinical and technical researchers, while high performance and integration into the clinical routine are not part of its scope. A first basic version of the RFA Guardian software has already been developed by the consortium in the EU FP7 project IMPPACT^34^. However, this predecessor did not support GPU acceleration.

The above software environments either require large supercomputers, which are not practical in the clinical environment, or lose accuracy as they omit patient-specific parameters. The proposed RFA Guardian rectifies these deficiencies as it has been developed to predict the treatment outcome in a couple of minutes while respecting all relevant patient- and device-specific parameters.

### The RFA Guardian

A comprehensive, user-friendly planning and simulation application for RFA needs to address the following scenarios within a single, local environment: (1) pre-interventional (PrI) simulation, which includes parameter space sampling for uncertainty estimation, prospective prediction of tumor coverage and correlated optimization of treatment cycles and access paths, (2) peri-interventional (PeI) simulation for the confirmation of parameterization in advance of treatment, reacting to unforeseen circumstances and concurrent simulation and treatment for confirming success, and (3) post-interventional (PoI) simulation aiding in training and education, examination of outcomes in advance of patient monitoring, and investigating failed treatment.

For easy integration into the clinical workflow, it is necessary that such a comprehensive application performs all computations in a fast and accurate manner. Moreover, the application needs to efficiently exploit the available standard hardware on a single high-end PC, instead of an expensive, often external, distributed computing cluster. Finally, all mentioned scenarios and their respective algorithmic requirements should be included in a single, user-friendly interface.

The presented RFA Guardian employs a generalized medical workflow, capturing the commonalities observed in four European clinical sites. It provides means for patient-specific modeling, faster-than-real-time simulation on the graphics processor (GPU) and advanced visualization for validating real and simulated treatment. We focus on exploiting all capabilities of a single PC for optimized performance, both in terms of time and accuracy. While automatic access path optimization is not part of the implemented feature set, the overall flexibility and high performance of the RFA Guardian allow for a straight-forward extension in this direction in future work.

IRs of any experience level can benefit from using the RFA Guardian for planning, validation and investigation. Moreover, its modularity also provides a testbed for device vendors and researchers.

## Methods

**Figure 1:**
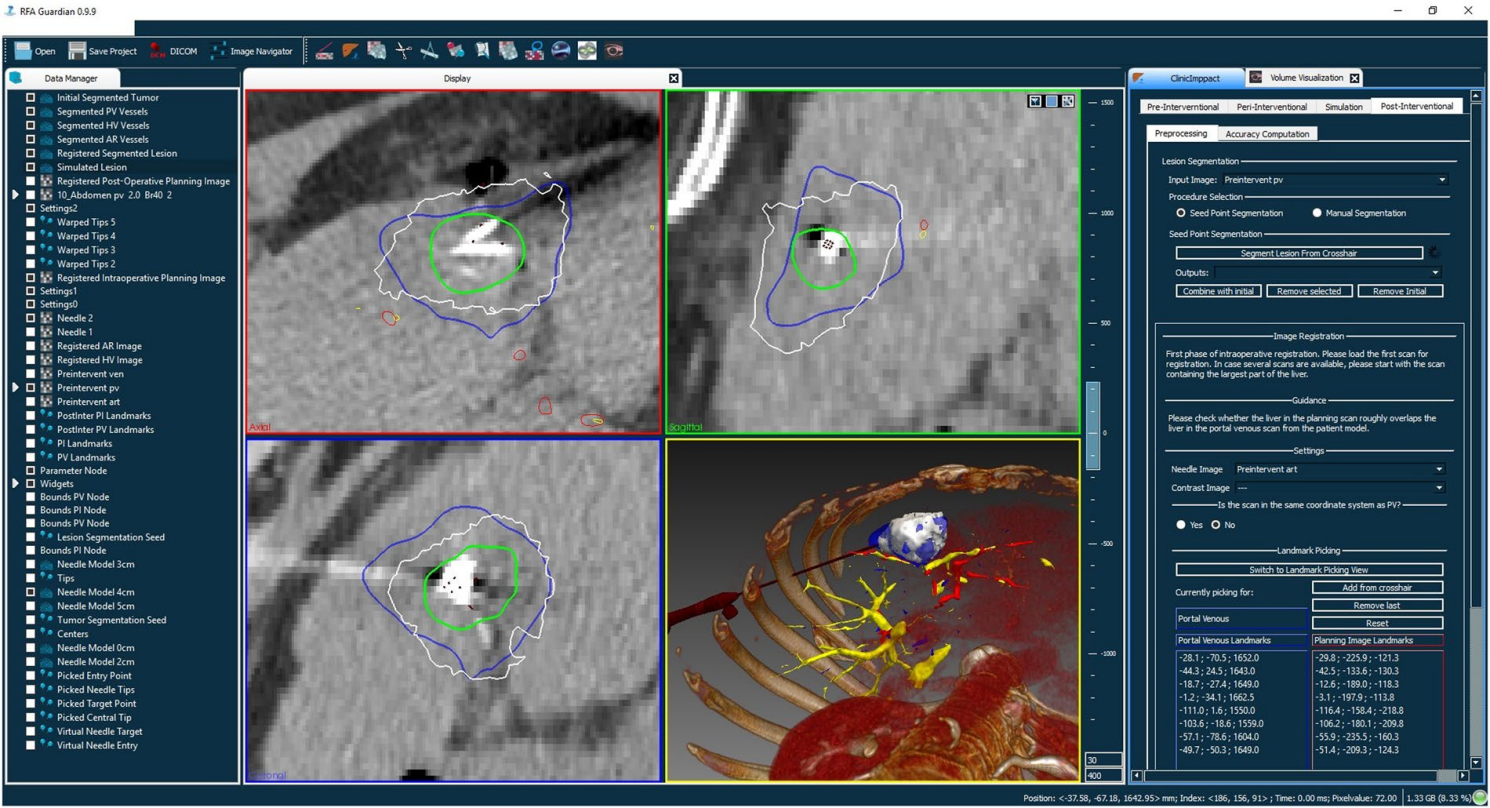
The RFA Guardian interface, showing a case from the pre-clinical evaluation. The left-most part is the data manager, a collection of all data loaded and generated during the workflow. The large central area is reserved for visualization, containing three orthogonal slice viewers and a 3D representation. The right part contains the control elements for navigating through the technical wokflow.

The RFA Guardian is an integrated, single PC application, combining a considerable number of image processing, biomechanical simulation and visualization algorithms into a single ergonomic interface. The focus on high computational performance enables fast and accurate simulation of the RFA treatment in PrI, PeI and PoI scenarios. The basic GUI (Fig. 1) resembles standard radiological workstations and includes an extensive set of features for different use cases with simple means for manipulation. Multiple, adjustable views for inspecting patient data occupy the largest part of the interface (Fig. 1, center). Additional key elements offer management and adjustment of input and generated datasets (Fig. 1, left) and control elements for executing tasks in the technical workflow (Fig. 1, right). Before detailing the feature set of the RFA Guardian, the upcoming section introduces the intended use cases resulting both from clinical and technical demands.

### Use Cases

From the clinical perspective, several scenarios for simulation of RFA treatment of liver tumors arise. Prospective prediction of the effects of certain parameterizations or exploring the parameter space and the resulting ablation zones can be critical tools in planning treatment. For education or evaluation purposes, retrospective simulation of real treatment configurations is important. The RFA Guardian aims at covering as many scenarios as possible with its comprehensive feature set. Table 1 shows a brief overview of the use cases and which features they use, while the upcoming subsections provide a bit more detail on the different intentions.

**Table 1:** Features of the RFA Guardian contributing to the various usage scenarios. x and o denote mandatory and optional modules, respectively.

Step	Pre-Interventional		Peri-Interventional		Post-Interventional		
	Pr-1	Pr-2	Pe-1	Pe-2	Pe-3	Po-1	Po-2
Model	x	x	x	x	x	x	x
Virtual Needle Placement	x	x	x	(x)	(x)	(x)	(x)
Real Needle Segmentation				(x)	(x)	(x)	(x)
Needle Registration			o	(o)	(o)	(o)	(o)
Simulation	x		x	x	x	x	x
Simulation Parameter Sampling	o	x	(o)	(o)			o
Real Lesion Segmentation					x	x	x
Real Lesion Registration					x	x	x
Statistical Evaluation	o	o	x	x	x	x	o
Visualization-Guided Evaluation	o	o	o	o	o	o	o

Items in brackets highlight branching workflows, mostly resulting from the underlying data. Especially the needle definition depends on whether the real needle is visible and requires registration into the PrI model. Using parameter space sampling, on the other hand, depends on whether tissue parameters can be precisely estimated or measured for a given case.

#### Pre-Interventional Planning (Pr-1)

This scenario aims at finding appropriate access paths and positioning for the needle before the intervention. Prospective simulation allows the IR to estimate the outcome in a safe scenario and to translate the insight into a real treatment procedure for that patient. This also includes the parameterization of the device, e.g. selecting appropriate heating protocols. From the perspective of the day-to-day clinical routine, this is probably the most important scenario, since it reinforces the current planning routine that rarely relies on accurate prediction software.

#### Pre-Interventional Parameter Space Sampling (Pr-2)

This is an extension of the planning procedure Pr-1 for cases where estimating tissue parameters or accurate reconstruction of planned needle positions are difficult. In such situations, generating an ensemble of simulations with slightly varying parameterizations can help the IR to estimate the variance of possible treatment results. For example, if no navigation systems aid the IR in faithfully reproducing needle positions are available, simulating with multiple, slightly varying needle positions can provide a good estimation the possible range of results.

#### Peri-Interventional Prospective Planning (Pe-1)

Besides PrI simulation, the RFA Guardian is the first of its kind that could enable simulation during actual treatment in the clinical routine due to its high performance simulation. While the patient is under general anesthesia, the IR can place a virtual needle in PeI images, register it into the PrI model and predict the outcome. This parameterization could, for example, serve as input into a navigation system which faithfully reproduces the positioning.

Moreover, a limited number of iterations for parameter space sampling is feasible within a reasonable timespan.

#### Peri-Interventional Prospective Confirmation (Pe-2)

Similarly to Pe-1, the IR can segment and register an already placed, real needle prior to treatment and use this parameterization for simulation. In case the resulting predicted coagulation area is unsatisfying, the IR can still adapt the treatment plan. This scenario, where treatment and simulation run in parallel, is currently being investigated in a clinical trial^35^.

#### Peri-Interventional Retrospective Validation (Pe-3)

The final shape and size of the coagulated region is ambiguous up until about one month after treatment. Simulating the treatment concurrently with the actual ablation, allows the IR to visualize the final result *in-situ* and perform minor corrections, such as additional heating cycles, during treatment.

#### Post-Interventional Retrospective Validation (Po-1)

This use case is similar to Pe-3, but lacks the option to react to possible shortcomings *in-situ*. Still, if the IR detects issues retrospectively, they can decide to adapt monitoring intervals or arrange additional treatment. Further, we believe that this can be exploited in educating inexperienced IRs.

#### Post-Interventional Evaluation (Po-2)

In the worst case of an unsuccessful treatment, retrospective evaluation of error sources can provide additional insight, both for education and future cases. For instance, the user can trace the effects of the power deposition and reason on the cause for failure.

### Technical Workflow

In the clinical routine, the workflow for CT-guided RFA treatment follows a rather straightforward path. First, the IR in charge plans the treatment according to available patient data, e.g., using diagnostic imaging. The intervention plan includes the number of required heating cycles with their respective parameterization and needle positioning. During the intervention, the IR implements the planned procedures step by step and at the end of the intervention checks size and shape of the coagulated region on ceCT images. After treatment, patients undergo follow-up imaging at regular intervals to detect potential (local) tumor recurrence.

**Figure 2:**
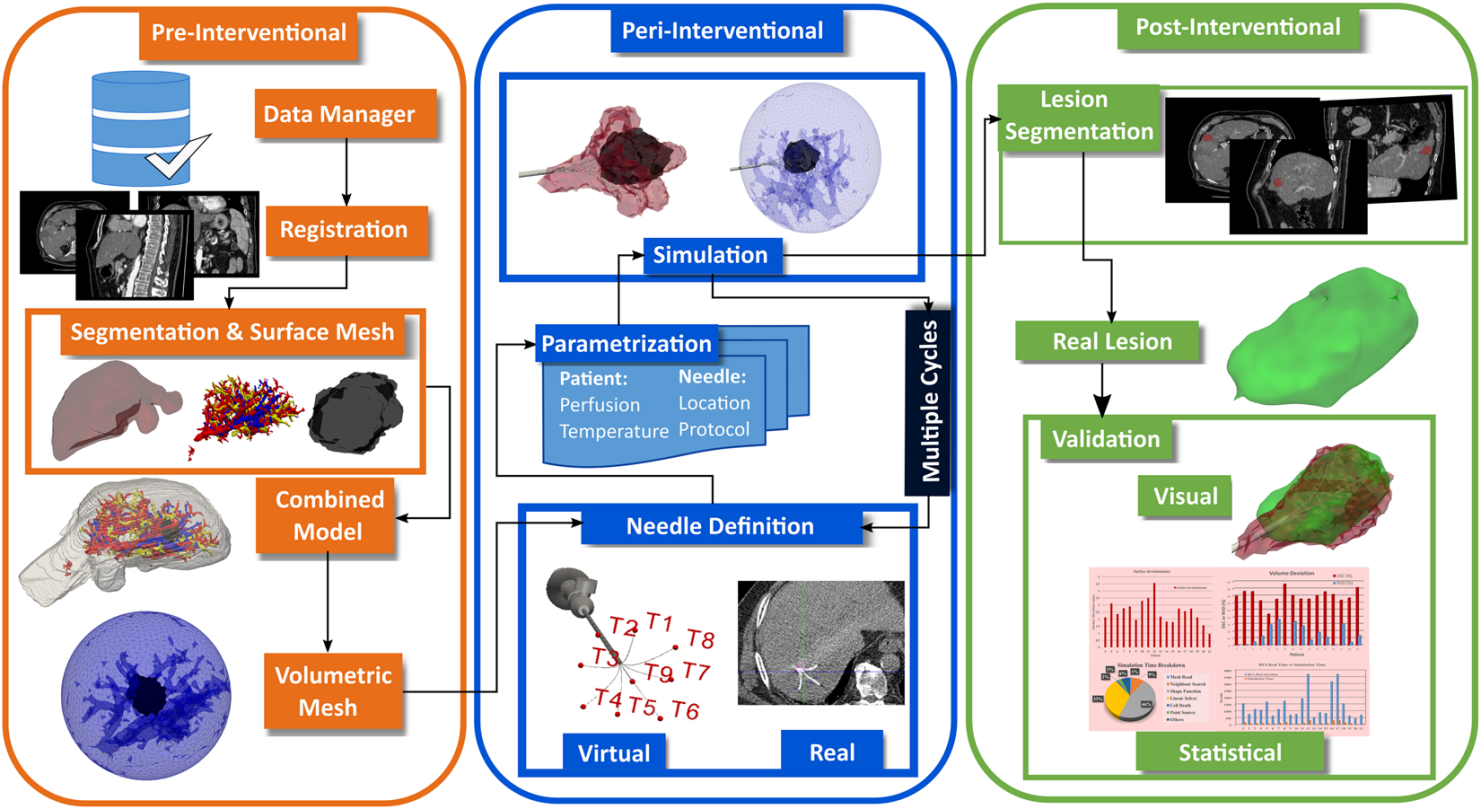
The technical workflow of simulation in the RFA Guardian. When considering the clinical procedure, three distinct phases assert themselves: In the pre-interventional stage, diagnostic scans contribute to generating a patient-specific anatomical model and potentially allow inference of parameters relevant for the simulation. This model allows for high-performance simulation in the peri-interventional phase with user-defined parameterization of the needle and generator. Finally, the post-interventional phase serves for validation purposes and potential analysis tasks for optimizing the treatment in advance.

The RFA Guardian further generalizes the workflow to aid the IR during the following three phases: (1) The Modeling Phase, for generating a patient-specific model comprising of anatomic structures and tissue-related parameters out of ceCT data; (2) The Simulation Phase, for accurate and quick estimation of the outcome of one or multiple treatment cycles, incorporating patient- and device-specific parameters and (distinct) needle positions. In addition, the RFA Guardian also provides parameter space sampling methods for mitigating uncertainties, e.g. arising during data collection or needle placement; (3) The Validation Phase involving quantitative assessment of treatment success, as well as advanced visualization for more in-depth analysis. Figure 2 provides an overview of the relationship between technical tasks and the medical workflow.

### Modeling Phase

The Modeling Phase serves as an initial stage for fusing patient information into a single model for simulation. Since patient-specific anatomy plays a significant role in the precise prediction of RFA^12,13,36^, we incorporate fast and accurate image processing methods for segmentation and registration and allow for manual correction. Patient- and device-specific parameters, which the RFA Guardian accepts through its interface, complete the patient-specific model.

The first step is automatic segmentation of the liver^37^ from a PrI ceCT image. The segmented liver capsule delimits the region for computation, preventing unnecessary computations in remote locations, and thereby increases the performance. It also serves as an important parameter for registration throughout of the RFA Guardian.

Since vascular structures in proximity to a RFA probe strongly influence the heat diffusion, the RFA Guardian consecutively registers multiple ceCT PrI images into a common coordinate system using fully automatic procedures. Although optional, optimal accuracy can only be achieved by using a ceCT image each for the arterial, portal venous and hepatic phases during processing. For the remainder of the paper, we assume availability of all ceCT phases.

Usually, all ceCT scans are recorded with minimal patient movement. However, motion correction^38^ showed to be mandatory and was therefore implemented to compensate anatomical discrepancies between individual phases due to breathing. From these registered images, the vessel trees are then automatically segmented^39^.

Due to the considerable computational demand, these steps are comparably time-consuming. To avoid constant attendance of the user, most of the procedures have been automatized in a feed-forward pipeline. After assigning loaded ceCT images to their corresponding phase, a single mouse click is sufficient for computing this pipeline. The set of segmentations (liver outline and vessel trees) resulting from this pipeline forms a registered patient-specific anatomical model. For difficult cases, the RFA Guardian additionally offers tools for manual correction of non-optimal results in each step.

The tumor segmentation completes the patient-specific anatomical model. Unfortunately, automating this step is barely feasible. Different tumor types expose varying tissue parameters and arbitrary localization, so we resort to a semi-automatic region growing approach^40^ using user-defined seed points. For tumors with heterogeneous tissue density, multiple seed points may be required. The user can choose to combine or neglect the single segmentation parts resulting from multiple seed points. Again, for very difficult cases, the RFA Guardian provides manual refinement tools. Moreover, to avoid an additional registration step, the seed points are defined directly in the registered ceCt images from the previous automatic pipeline.

The simulation domain is then defined by creating an optimized volumetric mesh for finite element (FE) simulation^41^, centered at the tumor. All previously created registered segmentations are fused into a single, space filling, tetrahedral mesh. During RFA treatment, the effect of heat deposition diminishes with distance from the probe, which is typically inserted close to the tumor. These observations enable optimizations of the simulation domain: Firstly, it is restricted in size. Since standard RFA protocols in the liver exhibit maximum coagulation diameters of 5 cm, the overall simulation domain is limited to a sphere with 6 cm radius around the tumor. Tissue beyond this border typically does not exhibit considerable influence on the heat distribution due to the large distance. Secondly, adaptive resolution techniques focus higher accuracy into critical regions. Especially the interaction around the tumor surface and near large vessels is important and requires a high FE mesh resolution. In mostly homogeneous tissue, the resolution can be lower and, consecutively, decreases the computational demand for these portions.

### Simulation Phase

The FE mesh resulting from the modeling phase forms the domain for the simulation. The workflow of this stage splits up into several branches, depending on the use case. In a nutshell, the basic steps comprise definition of device-specific and patient-specific parameters as detailed below.

#### Needle Definition

The user can choose between placing a virtual needle model, or segmenting and registering a real needle from PeI CT imaging. The choice depends on the specific scenario and available data, but both ultimately yield comparable input parameters.

Real Needle: From a PeI CT image, the user can segment and register a real needle. Both prospective and retrospective scenarios profit from accurately reconstructing the geometry, e.g. of umbrella-shaped probes. These often feature mechanically changeable extensions for different ablation sizes. We devised a simple, yet accurate and effective workflow for determining these needle geometries: In a patient image, the user manually selects the needle tip and a point along the shaft for simple models, and additionally the single prongs of extensible umbrella-shaped needles. For varying extensions that are not recorded in images, a simple interpolation (or extrapolation) from the input geometry is sufficient to accurately reconstruct the geometry.

The consecutive registration of this geometry into the PrI model employs the following strategy. First, the user needs to resolve large spatial deviations (e.g. occurring due to lateral positioning of the patient, or different offsets of different scanners) via picking a set of matching landmarks in the PeI images, as well as the PrI needle image. To simplify this process, the RFA Guardian provides axial and sagittal views of both images side-by-side. This allows to accurately localize and select landmarks in both images concurrently.

Using these landmarks, the RFA Guardian enforces a fast rigid registration method. In many cases, this optimally matches the images from PrI and PeI scanning sessions. However, RFA needles exhibit a certain flexibility, possibly leading to deformation and deviation from the optimal shape. Moreover, previous partial liver resection can complicate the process. Therefore, rigid registration can lead to insufficient accuracy, raising the need for an additional deformable registration method^38^ to compensate for local deformation. The previously defined landmarks already provide a good initialization for this method and, in most cases, further improve the registration accuracy.

Virtual Needle: Virtual needle placement is relevant for prospective planning, but can also act as a fallback for other scenarios, e.g. if the needle geometry is unavailable or obstructed in PeI images.

The user can place a virtual needle either directly in the PrI simulation domain, or relative to a PeI image. Generally, the virtual needle geometry can be defined using the intersection point between the needle tip and the tumor and a trocar point, which is any point along the needle shaft. However, more intricate needle models, for instance from Boston Scientific and RITA, exhibit a more complex geometry. This additionally requires parameterizing the rotation around the axis defined by the trocar and intersection. While manually replicating the exact positioning of the virtual needle for a real patient can be difficult, many clinics nowadays routinely employ navigation systems. If the user places a virtual needle to fit the real model in a PeI image, the same registration procedures as for real needle identification apply.

#### Device-Specific Parametrization

Apart from the needle positioning, device-specific heating profiles play an important role in the simulation process. The heating profiles are vendor-defined procedures, comprising duration of heating, cooldown cycles, iteratively extending umbrella-shaped needles, target temperatures, wattage, and many more. Again, the most complex procedures result from the umbrella-shaped probes, e.g. from RITA devices. The RFA Guardian implements predefined sequences, as provided by the vendors, and let the user choose the appropriate protocol. Although standard presets for target temperatures and power emission are provided by the RFA Guardian, the user can also modify these to correlate to the settings used during real treatment.

#### Patient-Specific Parameterization

Besides device-specific parameterization, measuring or estimating tissue-specific values contributes to the overall prediction accuracy. Perfusion measurements for healthy and malignant tissue are nowadays often part of the clinical routine. Other parameters, such as specific heat capacity or thermal conductivity, can often only be estimated. Nevertheless, the RFA Guardian provides interface elements for injecting these values into the simulation in case they have been measured or can be estimated accurately.

#### Parameter Space Sampling

Often, uncertainties exist when simulating RFA treatment. These can result from difficulties in reproducing planned needle positions or when measuring patient-specific parameters. However, the high performance of the implemented simulation strategy enables generation of multiple predicted coagulation zones in reasonable time spans. If the user is uncertain of certain inputs, they can choose up to two parameters (*p*_1_, *p*_2_) to simultaneously vary with (#*it*_*p*1_, #*it*_*p*2_) iterations, respectively. This results in *it*_*p*1_ * *it*_*p*2_ distinct configurations which are then simulated distinctly. The variable parameters include tissue perfusion, tumor perfusion, specific heat capacity and thermal conductivity, For these, the user specifies the variation range, number of iterations, and whether to randomly sample within this range, or linear interpolate is used.

The final variable parameter is the needle geometry. For simple models, typically only the tip and shaft orientation are of interest. For complex shapes, however, the single prongs are of particular interest. The flexibility of these instruments can lead to varying distances between the prongs, and, consecutively, modify the energy distribution. Here, the user can choose how many of the prongs should be considered for variation and the deviation range *r* (in *mm*). These points, including the needle tip, point on the shaft and potentially the single prongs, have their initial values set to the result from the Needle Identification and registration procedures. Variation is then achieved by moving the single positions in random directions within a sphere of size *r*, centered at the original point. Again, the user can choose how many iterations and, consecutively, different needle geometries, should be considered for the simulation.

**Figure 3:**
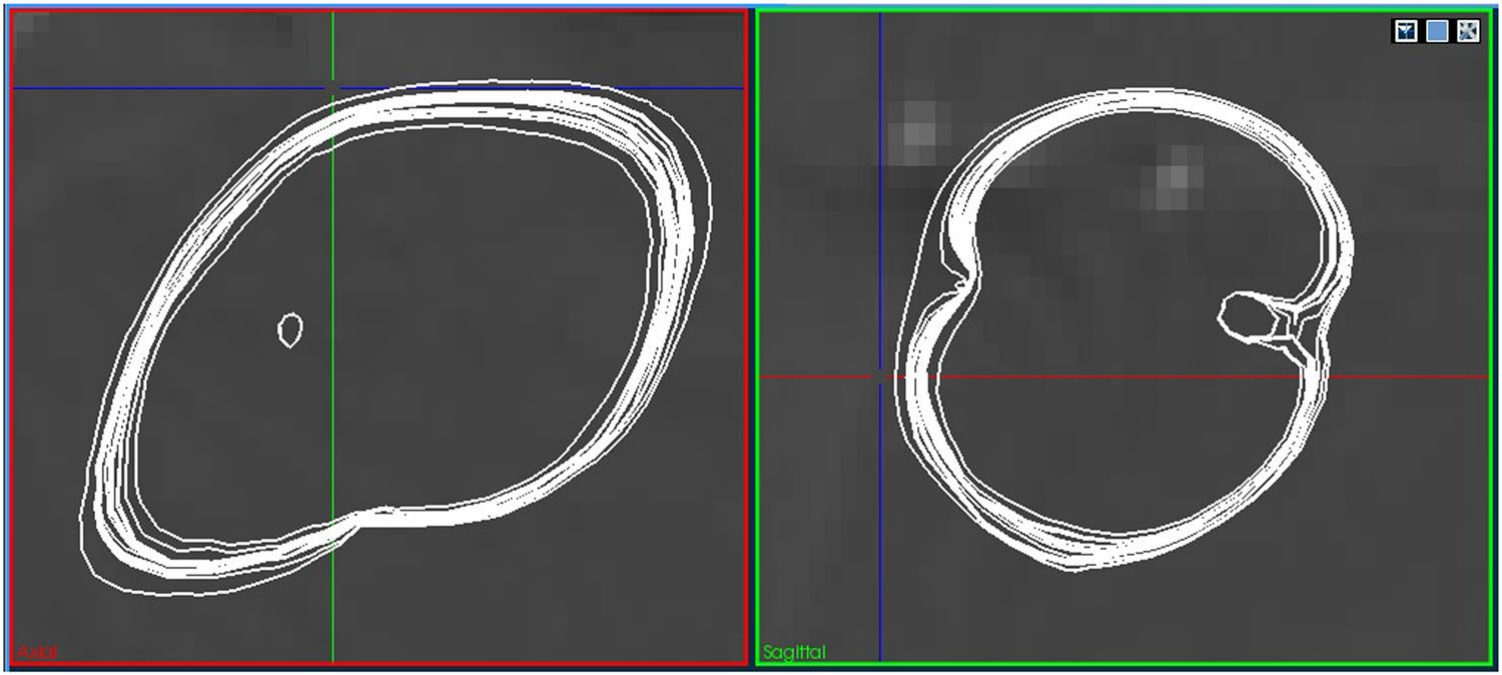
A simulation ensemble generated from the parameter space sampling routine. This particular set contains 25 simulation results while varying perfusion of tumor and tissue concurrently, with 5 linear variations each. Even small uncertainties in measuring the parameters can already have a considerable impact on the outcome. However, the parameter space sampling allows the user to estimate the expected range of results.

**Figure 4:**
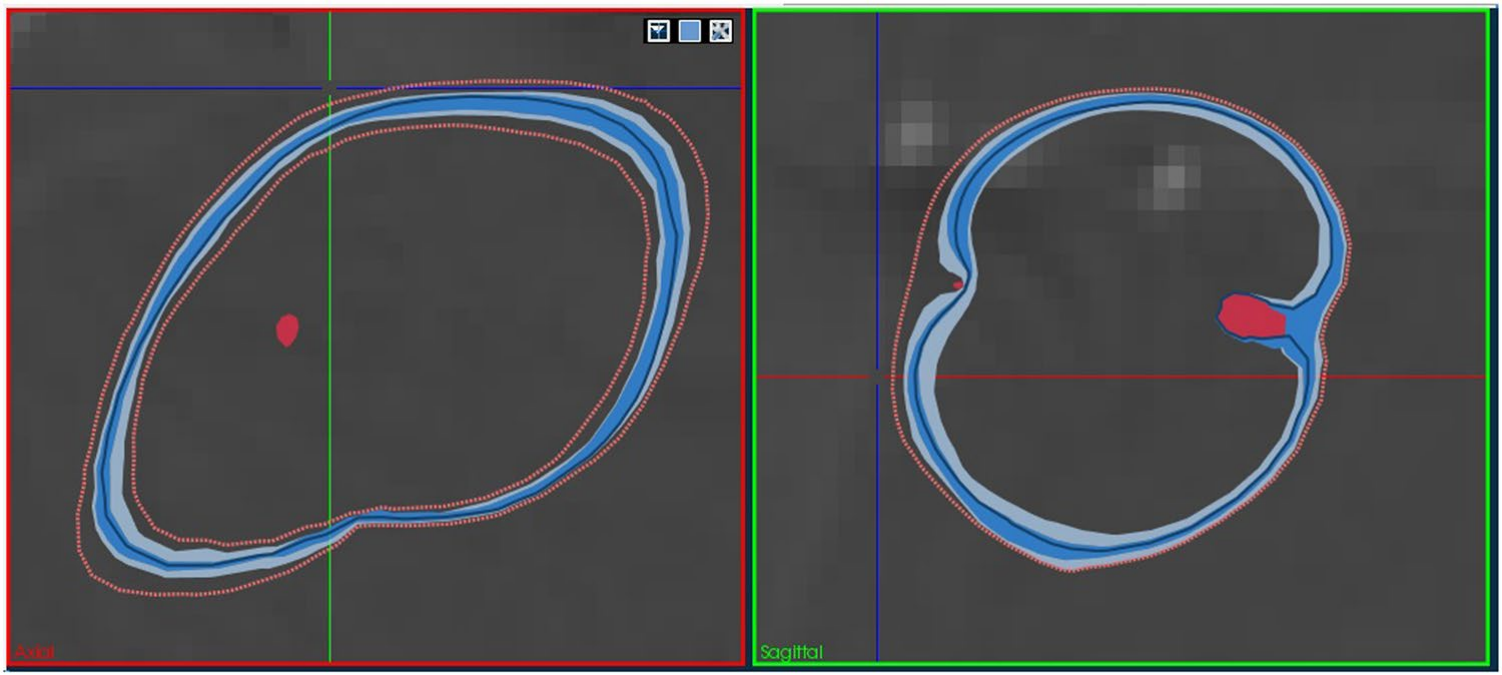
The same ensemble as in Fig. 3, but visualized with Contour Boxplots^42^. The simplification and consecutive condensation of information into a median (dark blue) and respective variance (medium blue and light blue), in conjunction with outliers (orange) and vessels (red), provides more insight into the behaviour of the lesion under varying parameters. Using this technique, the user can reason whether, e.g. the vessel is likely to be coagulated or survive. Factors like this can be critical in treatment planning, but are often overlooked in planning applications.

The resulting *it*_*p*1_ * *it*_*p*2_ predictions are then visualized for closer inspection by the user. Standard visualization techniques (Fig. 3) fail to provide the necessary insight into the resulting simulation ensemble. Especially the density of results is hard to determine in certain areas, leading to difficulties in predicting the outcome. Hence, the RFA Guardian implements a variation of Contour Boxplots^42^ to provide the user with an overview of what variations to expect. Figure 4 presents a simplified version of the same ensemble, highlighting the median, variance, outliers, and critical vessels. In combination with toggling certain parameter ranges on/off, the user can analyze the range of results to expect within the provided parameter variation and, potentially, optimize treatment parameters in advance.

#### Simulating Single and Multiple Cycles

After defining the simulation parameters, the user initiates the GPU-based computation. For any simulation scenario, the power deposition is computed using Gaussian distribution^25^ around the needle tip. This produces temperature distributions according to Penne’s bioheat equation^43^. A temperature-dependent three state cell death model^44^ is used to predict the cell death likelihood. The predicted coagulation volume is then extracted as described in literature^41^. A massively parallel GPU implementation^41^ provides the desired computational performance. For instance, 5 cm ablation protocols take roughly 3 minutes to compute, while the corresponding real ablation protocol takes at least 15 minutes.

The visualization section of the RFA Guardian continuously displays the outline of the coagulated area during this process. This real-time lesion tracking can provide valuable insight for the operator, e.g. when observing the behavior in critical regions.

After computation finishes, the simulation module goes into an idle state, waiting for additional input for further ablation tasks. After executing the first standard protocol, the user can perform additional heating in the same needle position with customizable duration, or conduct additional protocols using different needle positions. This is often the case in clinical routine, where tumor size or shape may demand multiple protocols. Although, usually, the number of cycles is reasonably low, unlimited, arbitrary combinations of standard and additional heating procedures are possible. For convenience, parameterization of each cycle is stored. This enables the user to replay each step of the simulated treatment and explore different strategies.

### Validation Phase

In general, planning RFA prospectively requires accurate and reliable predictions. In the Validation Phase, several techniques catering to the different usage scenarios aid the user in evaluating real or simulated results with a number of computed metrics. From a technical perspective, this can help in assessing the quality, while, from a clinical perspective, this can improve the trust in prospective planning based on simulation. The tasks basically boil down to comparing meshes and evaluating the distance and overlap between them. In case of comparing real and simulated coagulation zones, the distance should be minimized and the overlap should be high. When comparing simulated or real treatment to a tumor, investigating the safety margin around the tumor is critical, which implicitly also covers overlap measurement.

The RFA Guardian provides several metrics from literature^25,41,45^ for comparing two meshes. The indicators can be subdivided into two groups. For one, the distance between the meshes, condensed into the average absolute error (AAE) and minimum/maximum 3D distance are important. Additionally, volumetric measurements, including the relative volume difference (RVD) and sensitivity are provided.

In many cases, validation additionally requires registration and segmentation of real treatment results. The real lesion shape and size only manifests in the ceCT control one month after RFA treatment, which the user needs to segment from PoI images and consecutively register into the PrI model.

#### Real Treatment vs. Tumor

While this use case plays a lesser role in the RFA Guardian, it is still possible to evaluate real treatment within the application. Comparing a tumor, segmented from PrI images, with a segmented and registered real lesion, yields information about treatment success, e.g. whether the required safety margin is respected.

#### Simulation vs. Tumor

For prospective planning, the RFA Guardian enables comparison between a simulation outcome and the tumor. Similar to real treatment, the most important metrics to inspect are AAE and minimum/maximum distance between the predicted coagulation zone and the segmented tumor. In this scenario, registration and segmentation are obviously not necessary and the user can simply select the simulation result as input for the metrics computation.

#### Simulation vs. Real Treatment

During development, this was the most important use case. For comparing a simulated lesion with real treatment, segmenting the coagulation zone from a PoI image and registering it into the PrI simulation domain is often necessary. However, the significant time span between PrI images and PoI follow-up leads to considerably higher abdominal deformation compared to the short interval between PrI and PeI phase. Further, the region around the coagulated area tends to shrink over time, an effect that appears to be strongest in non-cirrhotic livers. These factors lead to the definitive need for deformable registration procedures to match the patient anatomy, similar to the PeI needle image registration.

The user then compares the registered, segmented real lesion with the simulation result. In this case, a few more metrics are relevant. Besides AAE and minimum/maximum distance between the meshes, additional volumetric considerations are important. During preliminary evaluation of the RFA Guardian, an intervention was considered to be successful^41^, if the RVD <20%, sensitivity >80% and AAE is below 3 mm.

#### In-Depth Simulation Investigation

In case the IR is not satisfied with the simulation result, determining the specific problematic areas in state-of-the-art software typically requires tedious, time-consuming manual measurements and slice-by-slice evaluation. To remedy these issues, the RFA Guardian provides advanced visualization techniques^46^. The implemented approach provides three consecutive stages of evaluation. While the algorithm is capable of catering to a wider range of scenarios, the RFA Guardian mainly exploits direct comparison of a simulation result to a given segmented tumor. The first stage enables fast evaluation whether the necessary safety margin (typically, 5 mm) between coagulated region and tumor is satisfied by a simulation result. The visualization displays a thick, color-coded rim at the outline of the coagulated region in 2D. The color coding categorizes portions of the outline into definitely failed (*distance* < 4.5 *mm*), critical (4.5 *mm* < *distance* < 5.5 *mm*) and probably safe (*distance* > 5.5*mm*). While scrolling through the slice stack, the user gets a quick overview of the successfulness of a parameterization. Moreover, the algorithm provides multivariate visualization techniques for in-depth analysis of the simulation domain. The user can choose two parameters that are additionally visualized. The first variable is encoded using adjustable, colored iso-bands. These are similar to iso-contours, but additionally encode gradients in the underlying field via a custom width with a smooth fall-off at the margins. The second variable is customly categorized and displayed using structural elements.

While the first technique provides a fast qualitative overview for the operator of the RFA Guardian and is applicable in the day-to-day clinical routine, the multivariate analysis caters more towards experts in the biomechanical simulation field.

## Implementation

All previously mentioned algorithms were implemented in C++, exploiting the open source MITK^47^ framework, which provides basic functionalities for medical workstations. The single modules rely on VTK (http://www.vtk.org) for visualization tasks, while all image processing algorithms have been implemented in the ITK (http://www.itk.org) framework. The FE mesh creation was implemented in the Computational Geometric Algorithms Library (CGAL http://www.cgal.org) and Gmsh (https://www.gmsh.info). The simulation procedures exploit massively parallel GPU acceleration via NVidia CUDA (http://www.nvidia.com/object/cuda).

The interface frontend implements predictive schemes to hide the complexity of the workflow during interaction and simplifies the usage of the RFA Guardian. Connected tasks were grouped into coherent interface blocks. Depending on both user interaction and available data, only the methods specifically relevant to the possible scenarios are enabled. The GUI comprises components of MITK, but futher extends the Qt (http://www.qt.io) library for specific tasks.

## Results

The RFA Guardian has been evaluated in a pre-clinical trial, especially in terms of its capabilities for PoI simulation and PrI planning. Data of previously treated cases, exposing heterogeneous imaging protocols, served as benchmark for testing. Further, the technical workflow has been optimized towards the final objective, namely PeI prospective simulation of treatment with the RFA needle in place. Since the clinical trial employing the RFA Guardian is still ongoing, final data for this objective is not yet available. Instead, results based on the PoI simulation of real cases are presented. These are analyzed in terms of time requirements per workflow step. Especially the simulation phase, including needle identification, needle registration and simulation itself, is time-critical for PeI simulation. Secondly, the predicted coagulated areas need to be accurate with respect to the results of real treatment. We received consensus for disclosing the data from five patients and provide them online along with a viewer application for reference at http://www.numa.ie/rfaguardian.

### Workflow Duration

During the evaluation phase, several IRs from four European clinics recorded the time required for each step of the whole RFA Guardian workflow while executing 10 cases from clinical practice retrospectively. Table 2 summarizes the records. In the following breakdown, the timings are annotated as (Average/Standard Deviation) in minutes.

**Table 2:** Timing for the different phases while using the RFA Guardian to simulate treatment in the post-interventional validation scenario (Po-1).

Step	Pre-Interventional		
	Automatic liver model	Tumor Segmentation	Volumetric Meshing
Average (minutes)	21,40	5,00	5,50
Standard Deviation	5,40	1,80	1,20
Sum (Std. Dev.)	28,5 (7,0)		
**Step**	**Peri-Interventional, 1st cycle**		
	**Needle Segmentation**	**Needle Registration**	**Simulation**
Average (minutes)	5,50	8,60	2,50
Standard Deviation	1,50	3,16	1,30
Sum (Std. Dev.)	16,6 (2,8)		
**Step**	**Peri-Interventional, 2nd cycle**		
	**Needle Segmentation**	**Needle Registration**	**Simulation**
Average (minutes)	5,50	4,80	1,50
Standard Deviation	1,00	1,90	0,50
Sum (Std. Dev.)	11,75 (2,25)		
**Step**	**Post-Interventional**		
	**Lesion Segmentation**	**Lesion Registration**	
Average (minutes)	3,90	6,30	
Standard Deviation	1,70	1,60	
Sum (Std. Dev.)	10,2 (3,3)		

While the pre-interventional steps are comparably time consuming, the peri-interventional simulation using placed needles (and the corresponding segmentation and registration tasks) are comparably fast. The statistics contain ten similarly structured cases from the clinical routine.

As expected, the automated part of PrI modelling is comparably time consuming (21.4/5.4). Tumor segmentation including manual correction (5.0/1.8) and volumetric meshing (5.5/1.2), however, are comparably fast and add little to the overall modelling duration (28.5/7.0). For simulating the first ablation cycle, the IR performed segmentation of umbrella-shaped needles (5.5/1.5) and landmark selection (5.8/1.8) for registering (2.8/1.36) the PeI images into the model before simulating (2.5/1.3). Moreover, four cases include a second ablation cycle, again requiring needle segmentation (5.5/1.0), landmark picking (2.8/0.9), registration (2.0/1.0) and simulation (1.5/0.5). In our opinion, the whole simulation procedure (16.6/2.8 first cycle; 11.75/2.25 second cycle) of the RFA Guardian is fast enough to justify the PeI prospective simulation goal and, in the future, effectively assist IR in the clinical routine. For completeness, the IR performed ablation zone segmentation (3.9/1.7), landmark picking (4.8/1.0) and registration (1.5/0.6) for images one month post ablation.

### Simulation Accuracy

**Figure 5:**
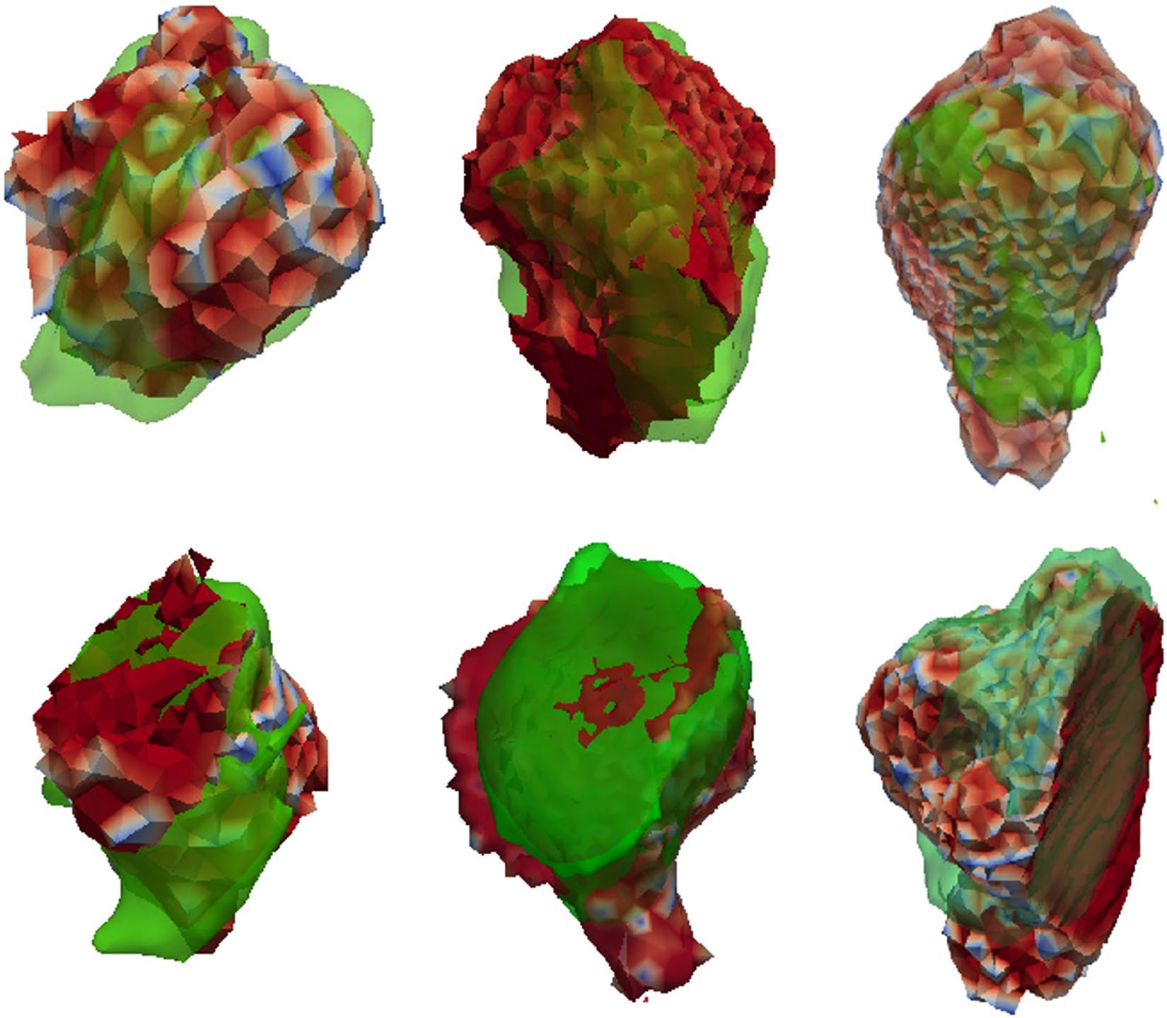
Visual comparison of six distinct simulation results (color-coded opaque mesh) and the respective segmented real lesions (green transparent surface). Despite non-optimal imaging conditions and protocols, the accuracy achieved during pre-clinical evaluation of the RFA Guardian is very promising.

As previously described, the success of simulation with the RFA Guardian is determined by evaluating how well the shape and size of the predicted lesion match those of the real treatment. On top of the temporal assessment, a multi center retrospective study has been conducted on 21 previously treated cases^41^. Statistical evaluation of these cases (Table 3) shows that the simulated lesion based on the RFA Guardian and the treatment outcome match quite well. Figure 5 shows a few comparisons between simulated and real coagulation areas, also exposing a good visual match.

**Table 3:** Comparing simulation results obtained in the RFA Guardian versus real treatment for 21 cases.

Metrics	DSC(%)	RVD (%)	SN (%)	PPV (%)	AAE (mm)
Mean	70,03	13,77	69,70	71,73	2,44
Standard deviation	9,37	12,96	10,94	12,00	0,84

Despite imaging procedures and parameter acquisition not being optimized and heterogeneous, we still achieve high accuracy. The table lists Dice Score (DSC), Relative Volume Difference (RVD), Sensitivity (SN), Positive Predictive Value (PPV) and Absolute Average Error (AAE).

For five of these cases, the results were below our expectations. Special difficulties have been observed during evaluation of tumors in non-cirrhotic livers. There appears to be a certain amount of post-ablation shrinkage in the comparably soft liver tissue for these patients. This induces considerable errors in the deformable registration procedure. For reference, we cross-validated these cases using a straight-forward rigid registration that only considers the lesion outlines and disregards the surrounding tissue. In some of these cases, we could indeed confirm the liver shrinkage as the source of the error. For the remaining cases, however, we could only trace back the remaining inaccuracies to the needle registration procedure. Even minor errors, such as moving a single needle tip closer (or into) a major vessel has a strong influence on the faithfulness of the prediction.

Another key factor that limits the achievable simulation accuracy is track ablation, performed to reduce the risk ot tumor seeding in the access path. While frequently used in clinical routine, simulating this procedure is infeasible since it involves slow, manual retraction of the probe without a fixed protocol. However, it is at the same time impossible to discriminate between portions induced by track ablation and normal treatment. Consecutively, the track ablation must also be considered in the accuracy evaluation and further diminishes our results.

**Figure 6:**
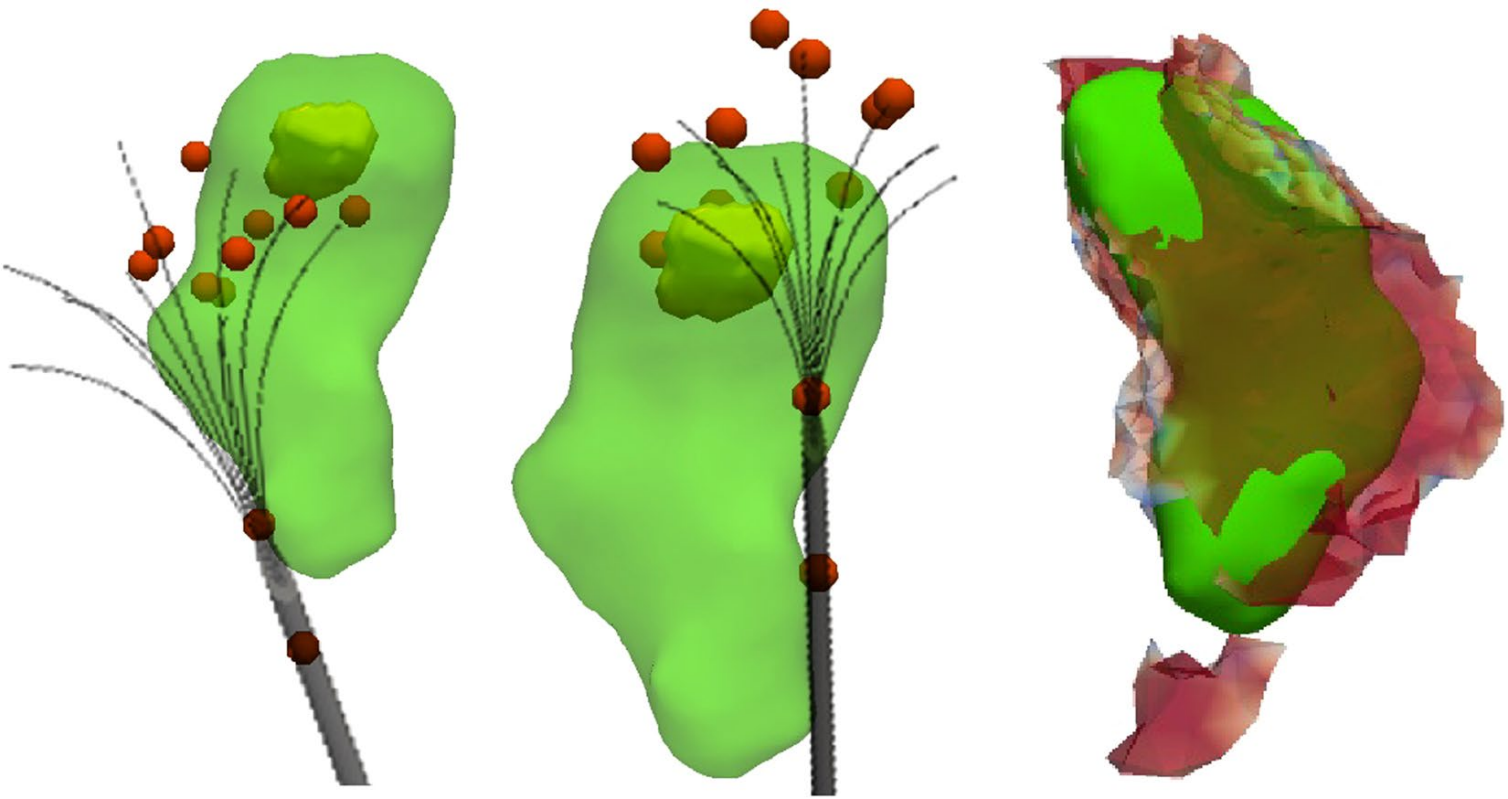
Visual comparison of an unsuccessful case with a tumor at the liver capsule. This particular case was treated with two consecutive ablation cycles with needle repositioning inbetween (left and center images). While the registration between real induced lesion (green) and tumor (yellow) seems appropriate, the registration of the needle geometry was not accurate enough due to severe tissue deformation at the liver capsule. The left and central figures shows an idealized needle model in gray, while the red spheres visualize the actual simulation input. These single tips resulted from segmentation and registration of the patient images for this case. The deviation from the optimal umbrella shape consecutively leads to a mismatch between simulated and real treatment (right), is below the necessary accuracy. However, such registration mismatches are typically easy to identify during the workflow and, consecutively, do not pose a risk for patients if observed carefully.

Figure 6 shows an especially difficult case. The tumor is comparably small (7 mm), which amplifies even small registration inaccuracies, and is located at the liver capsule. The mechanical force applied by inserting the needle leads to considerable tissue deformation, a factor we observed to be much larger for peripheral tumors compared to centrally located ones. Further, the liver is non-cirrhotic, implying larger deformations due ot the mechanical force. Additionally, the track ablation is comparably large with respect to the locally induced ablation. The combination of these factors lead to an unsatisfying result (DSC 69.07, RVD 81.28, AAE 4.57).

## Discussion

This paper presented the RFA Guardian, a comprehensive application for covering a broad range of use cases for simulating the RFA of liver tumors in the clinical routine. Using a uniform workflow, capable of branching towards handling exceptions or deviations from standard processes, the RFA Guardian provides a flexible framework that can be used for PrI planning, PeI prediction and evaluation and PoI analysis in the clinical routine.

Currently, the RFA Guardian is the center of a clinical trial^35^ that evaluates whether peri-interventional prospective simulation could be feasible in the future. To ultimately achieve this goal, the focus of the ongoing study is to record the time required for simulating treatment using a real needle position, segmented from peri-interventional images and registered into the patient-specific model. Of course, simulation accuracy is another critical end-point of the trial.

An early observation of this study is the need for two persons involved in the workflow: One person (an IR or technical assistant) is responsible for operating the software, while the IR can fully focus on the treatment. While moving from the pre-clinical trial to the clinical study, a considerable training effect has been observed for the peri-interventional tasks: While needle segmentation and registration, as well as simulation initially took up to 30 minutes per treatment cycle, the current average is at 16.6 minutes for the first and 11.75 for the second cycle (see Table 2). Discussions with the IR involved in the study suggest that the gain from accurate prospective simulations could outmatch the additional time requirement. Further, we believe that specialized training and increasing experience of the operator can further decrease the required time. Of course, future image processing and simulation techniques could also contribute to reducing the time requirements. Nevertheless, the IR revealed that integrating the RFA Guardian in its current state could provide sufficient benefit justifying the additional time requirements.

Since general anesthesia is part of the standard protocol of all clinical sites involved in the study, only using local anesthesia has not been considered. Feasibility of PeI prospective simulation under these circumstances definitely requires a separate trial.

The preliminary results (Section VII) show that the RFA Guardian is capable of fast and accurate prediction of intervention outcomes. This was only possible due to careful optimization of algorithmic and user aspects. Nevertheless, in some cases, the results are still below our expectations. In terms of accuracy, registration between the three imaging phases can be difficult. From a technical point of view, it would be highly desirable to move the complete modeling phase into the peri-interventional phase, which would lead to improved accuracy of image registration between the patient model and the needle images. However, this could mean excessive time under general anesthesia for the patient. Hence, the only way to improve this aspect is to explore other possibilities for registration between pre-interventional and peri-interventional images. When considering navigation systems, this is especially crucial for reproducing planned needle positions. However, these issues only occur in special cases, for instance in non-cirrhotic livers.

From a validation point of view, the considerable deformation observed in the post-interventional monitoring images, especially near the coagulated region, induces additional inaccuracies. In our opinion, this issue requires completely new image registration techniques that base on accurate analysis of tissue flexibility after RFA treatment.

Although currently purposely focused on a narrow range of device vendors, extending the RFA Guardian to additional, often more simple RFA generators is straightforward. Focusing the development of the RFA Guardian around RITA probes from the start was a deliberate choice. Considering that these introduce considerable complexity in terms of registration and computation, introducing simpler probes and generator models in future applications will be much easier than adapting the software the other way around. Further, extension to Microwave Ablation devices and their specific simulation setup is currently under investigation. This will provide capabilities for comparing different treatment modalities, allowing the IR to choose the best suited treatment for a patient.
